# The 16SrXII-P Phytoplasma GOE Is Separated from Other Stolbur Phytoplasmas by Key Genomic Features

**DOI:** 10.3390/pathogens14020180

**Published:** 2025-02-11

**Authors:** Rafael Toth, Bruno Huettel, Mark Varrelmann, Michael Kube

**Affiliations:** 1 Department of Integrative Infection Biology Crops-Livestock, University of Hohenheim, 70599 Stuttgart, Germany; michael.kube@uni-hohenheim.de; 2 Max Planck-Genome-Center Cologne, 50829 Cologne, Germany; huettel@mpipz.mpg.de; 3 Institute of Sugar Beet Research (IfZ), 37079 Göttingen, Germany; varrelmann@ifz-goettingen.de

**Keywords:** SBR, sugar beet, phylogeny, virulence factor, phytoplasma pathogenicity island

## Abstract

The syndrome “bassess richesses” is a vector-borne disease of sugar beet in Germany. The gammaproteobacterium ‘*Candidatus* Arsenophonus phytopathogenicus’ causes reduced sugar content and biomass, growth abnormalities, and yellowing. Co-infection with the 16SrXII-P stolbur phytoplasmas often leads to more severe symptoms and a risk of complete economic loss. This yellowing agent of the Mollicutes class had not been described before, so its differences from other stolbur phytoplasmas remained unanswered. The genome of strain GOE was sequenced, providing a resource to analyze its characteristics. Phylogenetic position was revised, genome organization was compared, and functional reconstructions of metabolic and virulence factors were performed. Average nucleotide identity analysis indicates that GOE represents a new ‘*Ca.* Phytoplasma’ species. Our results show that GOE is also distinct from other stolbur phytoplasmas in terms of smaller genome size and G+C content. Its reductive evolution is reflected in conserved membrane protein repertoire and minimal metabolism. The encoding of a riboflavin kinase indicates a lost pathway of phytoplasmas outside the groups 16SrXII and 16SrXIII. GOE shows a complete *tra5* transposon harboring orthologs of SAP11, SAP54, and SAP05 effectors indicating an original phytoplasma pathogenicity island. Our results deepen the understanding of phytoplasma evolution and reaffirm the heterogeneity of stolbur phytoplasmas.

## 1. Introduction

In the European Union, Germany is one of the largest producers of sugar beet (*Beta vulgaris* subsp. *vulgaris*), with 21,730 farms producing >29 million tonnes from approximately 364,000 hectares (WVZ, 2024). Today, German sugar beet growers are challenged by an outbreak of a bacterial disease called syndrome “basses richesses” (SBR), which now affects about 75,000 ha of sugar beet in Germany and is still spreading (unpublished data). When first described in Burgundy (France) in the 1990s, SBR was blamed for halving sugar beet growers’ incomes, underlining the importance of this disease until today [1]. SBR is associated with degeneration of the vascular system, yellowing and necrosis of old leaves, proliferation, formation of new lanceolate leaves, and a reduction of up to 5% of the absolute sugar content and up to 25% sugar beet biomass [1,2,3]. The causative agent of SBR is ‘*Candidatus* Arsenophonus phytopathogenicus’, a γ-proteobacterium [4,5,6]. In addition, co-infection of sugar beets with phytoplasmas in a small percentage of SBR-diseased sugar beets was noted early [4,5]. In contrast to ‘*Ca.* A. phytopathogenicus’, phytoplasmas are wall-less bacteria of the class Mollicutes. They are also obligate biotrophs and vector-transmitted. The γ-proteobacterium and phytoplasma are transmitted by *Pentastiridius leporinus* (Linné) to sugar beet during phloem sap-feeding from and to sugar beet. Both pathogens infect the phloem but histological effects differ between phytoplasma associated with cell necrosis and cell wall lignification, while ‘*Ca.* A. phytopathogenicus’ is in addition associated with the deposition of phenolic compounds in the lumen of phloem cells [5]. Further differences include the increased proliferation and reduction in biomass associated with phytoplasma infection of sugar beet [3].

**Figure 1:**
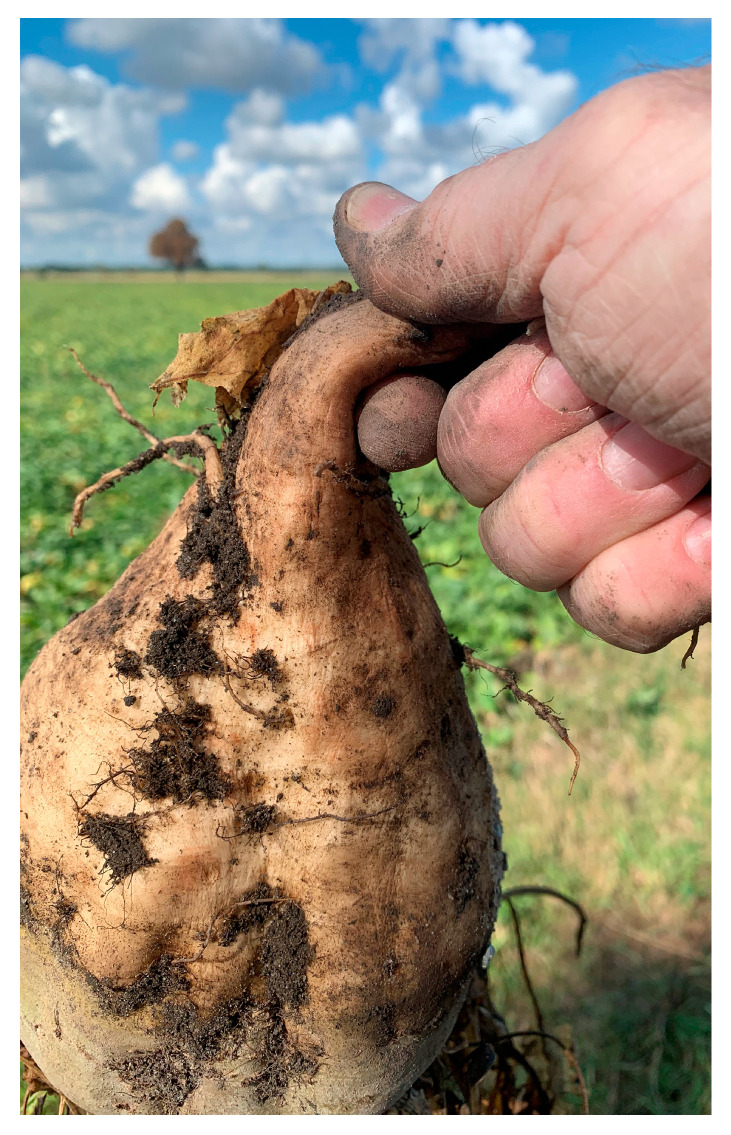
Rubbery taproot symptom on sugar beet in Elbe River Valley infected by ‘*Ca.* A. phytopathogenicus’ and 16SrXII-P phytoplasma.

In recent decades, the population of *P. leporinus* has increased dramatically following the exploitation of sugar beet as a host plant [7]. Vector fitness is also favored by the prominent crop rotation of sugar beet and winter wheat, which allows optimal development of pathogen-loaded larvae [8]. This could be an effect of short fallow treatments, taking into account a needed root system for sucking and the polyphagous nature of the larvae that can develop on different plant species [9,10]. This system, recognized early as problematic in France [3,8], is also blamed for the rapid spread of *P*. *leporinus* from 2008 until today in southwestern Germany and Switzerland [11,12,13]. In addition, optimal conditions, including high temperatures, favored the emergence of a second generation of *P. leporinus* in summer. Long vector presence in season and high prevalence increased the risk of infection of other important crops as it has been shown, e.g., for ‘*Ca.* A. phytopathogenicus’ infection of potato [14]. Of particular importance is the rapid increase of infection by both pathogens [9,15]. Little is known about the phytoplasma strains/groups involved in the infection of sugar beet in the first decades in France and Germany. Today, a new phytoplasma subgroup is driving the epidemic in Germany, discovered by investigating high sugar beet losses in the Elbe River Valley [15] and later also identified in Poland [16]. This phytoplasma strain showed a novel 16S rRNA gene sequence restriction profile that differed from other phytoplasmas [17,18,19] and was classified as a 16SrXII-P subgroup [15]. This new subgroup is part of the 16SrXII/stolbur group, which includes a wide range of vectors and hosts and species such as ‘*Candidatus* Phytoplasma solani’ (associated with bois noir disease of grapevine, potato stolbur [20], tomato stolbur [21], maize redness [22,23,24], and lavender decline [25,26]), ‘*Candidatus* Phytoplasma australiense’ (also causing grapevine diseases) [27], ‘*Candidatus* Phytoplasma japonicum’ (agent of Japanese Hydrangea phyllody) [28], and ‘*Candidatus* Phytoplasma fragariae’ (strawberry yellows) [29]. Sugar beet with double infection of the ‘*Ca*. A. phytopathogenicus’ and 16SrXII-P phytoplasma showed in combination with heat- and drought-stress rubbery taproot symptoms (Figure 1),which had previously only been described for ‘*Ca*. P. solani’ 16SrXII-A subgroup infections in Serbia [30]. However, the spread of the 16SrXII-P phytoplasma in the following years rapidly became dominant in several other regions, including southern Germany [31].

The first description of the 16SrXII-P phytoplasmas indicated that this subgroup is a ‘*Ca.* P. solani’ relative, but the number of available taxonomic markers was low [15]. The lack of genome information, especially complete phytoplasma genomes, is still striking in terms of impact. In 2024, 36 complete phytoplasma genomes were available (www.ncbi.nlm.nih.gov/datasets/genome/, accessed on 7 December 2024). They vary in size from 499–974 kb (www.ncbi.nlm.nih.gov/datasets/genome/?taxon=33926, accessed on 7 December 2024). The small phytoplasma genomes are characterized by reductive evolution [32] and encode a minimal metabolic repertoire resulting from colonization of nutrient-rich environments [33], as well as group-specific genes involved in vector and host interaction [34].

Until recently, only few genomic data were available for the stolbur group. Draft genomes were available for five strains of ‘*Ca.* P. solani’—strain 284/09 (FO393427), 231/09 (FO393428), SA-1 (MPBG00000000), STOL (JBFPNQ000000000), and ST19 (JBFSHS000000000)—and two strains for ‘*Ca.* P. australiense’—Tabriz.2 (JAINCS000000000) and Tabriz.4 (JAPFFB000000000)—as well as two complete genomes for the ‘*Ca.* P. australiense’ strains PAa (AM422018) and NZSB11 (CP002548). In 2024, the complete genome of strain GOE (CP155828) from the 16SrXII-P stolbur subgroup was determined [35] and three complete genomes of the ‘*Ca.* P. solani’ strains c1 (CP103788), c4 (CP103787), and c5 (CP103786) from *Convolvulus arvensis* (bindweed) and one from *Urtica dioica* (stinging nettle) namely strain o3 (CP103785) in Italy have recently been deposited in GenBank. This expanded database enabled us to perform a comprehensive analysis of strain GOE. Here, we revisited the distinct position of this 16SrXII-P strain in the stolbur group, performed a functional genome reconstruction with particular emphasis on host-dependent metabolism, and provided important insights into the virulence-associated phytoplasma mobilome.

## 2. Materials and Methods

### 2.1. Genomic Data

Comparative genome analyses within this work were based on all complete genome sequences of the 16SrXII stolbur group (NCBI:txid85632) including the complete genomes of the 16SrXII-P phytoplasma strain GOE (CP155828), the ‘*Ca.* P. solani’ strains c1 (CP103788), c4 (CP103787), c5 (CP103786), and o3 (CP103785) (NCBI:txid69896), and the ‘*Ca.* P. australiense’ (NCBI:txid59748) strains PAa (AM422018) and NZSB11 (CP002548) (NCBI:txid59748). All data were retrieved from NCBI (www.ncbi.nlm.nih.gov, accessed on 12 September 2024). Genomic benchmarks for comparison were assessed with the Artemis Genome Browser v18.2 [36] using the genetic code 11. If not otherwise stated, default settings were used.

### 2.2. Phylogenetic Comparison

Whole-genome phylogeny was assessed with average nucleotide identity (ANI) [37] computed with FastANI v1.3 [38]. In addition, sequence synteny analysis was conducted via Mauve v2.4.0 [39]. Whole-genome phylogeny analyses were performed with default settings.

To confirm the phylogeny on the whole-genome level, marker gene analysis was performed for phylogenetic assignment of strain GOE using the phytoplasma markers 16S rRNA and *tuf*. As a first comparison, the complete 16S rRNA gene sequences of GOE were used to infer identities with all other complete genomes of the stolbur group as well as with the reference strains STOL (AB639069) and 916/22 [15], obtained from BLAST analysis. Further, similar sequences for comparison were identified using the nested PCR amplicon of the primers R16F2n/R2 [40] and the fTufAy/rTufAy [41] amplicon from strain GOE and subjected as a query for a Basic Local Alignment Searching Tool (BLAST) analysis against the nucleotide collection (nt) databases from NCBI (accessed on 22 November 2024) [42]. Maximum target size was set to 500 and all filters and masks were disabled. BLAST outputs were inspected manually to select nucleotide sequences belonging to phytoplasmas of the stolbur group. In total, 254 nucleotide sequences were extracted for 16S rRNA and 46 for *tuf*, which were used for multiple sequence alignments, and which were calculated using the MUSCLE algorithm [43] within the Molecular Evolutionary Genetics Analysis (MEGA) software v.10.2.6 [44]. The evolutionary history was inferred via the maximum likelihood method within MEGA on default settings. Each tree was calculated with 1000 bootstraps to ensure statistical significance for cluster assignment. Sequences of ‘*Ca.* P. asteris’ strain M8 [32] were used as an outgroup taxon to root the calculated trees, and 16S rRNA taxonomy was additionally analyzed with iPhyClassifier [45] and used for 16SrXII subgroup assignment via in silico restriction fragment length polymorphism (RFLP) analysis. Default settings have been used except where indicated.

### 2.3. Ortholog Prediction

For the comparison of shared and unique features of the complete 16SrXII stolbur phytoplasma genomes, orthogroups of the deduced amino acid sequences were predicted with OrthoFinder v2.5.5 [46] and visualized with an upset plot using Intervene v0.6.5 [47] with default parameters. The number of shared and unique deduced amino acid sequences of GOE were extracted from the OrthoFinder results and summarized in a pie chart.

### 2.4. Functional Reconstruction

For the analysis of encoded metabolic pathways and membrane transport systems, InterproScan v5.68 build 100.0 [48], KEGG database release 109.1 [49], and MetaCyc database v28.0 [50] were utilized. The deduced amino acid sequence of riboflavin kinase of GOE was subjected to a BLASTP comparison for the identification of orthologs in other phytoplasmas which were then used to infer maximum likelihood phylogeny on the amino acid level using MEGA as described in Section 2.2. RibF of *Alteracholeplasma palmae* J233^T^ [51] was used as an outgroup taxon to root the calculated tree.

Secretome analysis was performed with Phobius v1.01 [52] to identify deduced amino acid sequences encoding either a signal peptide or at least one transmembrane domain or both. Predicted effectors of the strain were confirmed via SignalP v6.0 [53]. Structural alignments of the deduced amino acid sequences of the immunodominant membrane protein (Imp) were constructed with T-Coffee [54]. Structure prediction of Imp was performed via Alphafold v2.0 [55] within the Galaxy Europe server (accessed on 25 June 2024). In addition, maximum likelihood phylogeny was assessed for Imp on nucleotide and amino acid levels within MEGA as described in Section 2.2. Insertion-element (IS-element)-associated regions were predicted via ISEScan v1.7.2.3 [56], further analyzed via palindrome within European Molecular Biology Open Software Suite v6.6.0 to predict repeat regions, and visualized with clinker v0.0.29 [57] and Artemis v18.2 [36].

The genome-wide organization of strain GOE was visualized with DNA Plotter within Artemis v18.2 [36]. Unless otherwise stated, default settings were used.

## 3. Results

### 3.1. Genomic Benchmarks of Complete Stolbur Phytoplasma Genomes

**Figure 2:**
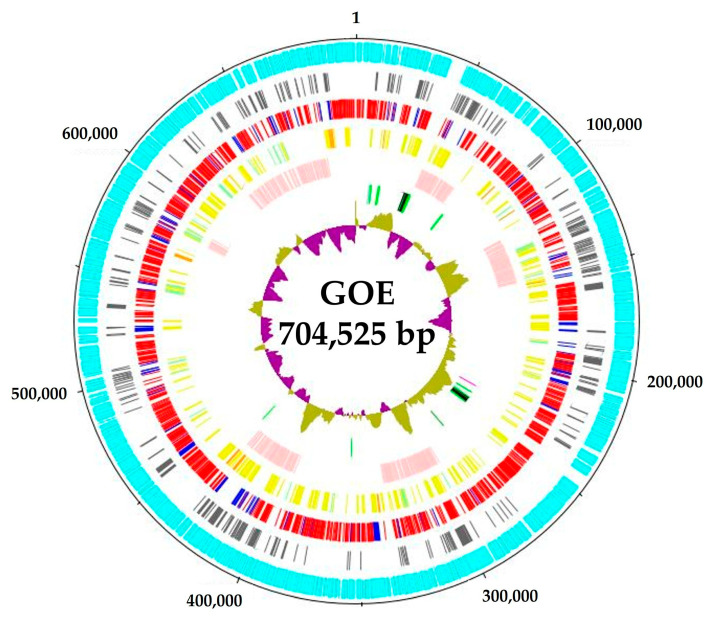
Genomic organization of the circular chromosome of strain GOE. Circular patterns (from outside): (1) (outer ring), scale in base pairs of the chromosome; chromosome regions including (2) (cyan), predicted protein-coding sequences; (3) (grey), hypothetical proteins; (4) (multi-colored), predicted protein-coding sequences: shared with all investigated genomes of the stolbur group (red), unique protein-coding sequences (blue); (5) (multi-colored), predicted membrane proteins: with only a signal peptide (pale green), only transmembrane domains (yellow), and both signal peptide and transmembrane domains (orange); (6) (pale pink) potential mobile unit (PMU)-like regions; (7) (multi-colored), RNAs: signal recognition particle RNA and RNase P RNA component class B (magenta), transfer RNAs (green), and rRNA operons (black), transfer-messenger RNAs (light blue); (8) G+C skew (olive and pink).

Strain GOE has a circular chromosome (Figure 2), like the other complete stolbur phytoplasmas. The chromosome size of the genomes ranged from ~704 kb of GOE to ~973 kb of ‘*Ca.* P. solani’ o3 (Table 1). Both also represented the outer limits of the analyzed G+C contents with 26.17% and 28.58%, respectively (Table 1).

With a total of 663 CDSs, GOE has the lowest number of CDSs with a moderate coding density of 0.941 genes per kb, while ‘*Ca.* P. australiense’ NZSb11 encodes the largest number with 1100 CDSs and the highest coding density with 1.146 genes per kb. All analyzed members of the stolbur group encode two complete rRNA operons typical for phytoplasmas [33], whereas the number of encoded tRNAs differs within the analyzed taxa, with 32 tRNAs for ‘*Ca.* P. solani’ and 35 tRNAs for ‘*Ca.* P. australiense’ (Table 1). All analyzed genomes harbor *ssrA* coding for a tmRNA [58] as well as the ncRNA genes *ffs* and *rnpB*. No plasmids were identified for GOE and the other complete genomes of the taxon ‘*Ca.* P. solani’, whereas for ‘*Ca.* P. australiense’, plasmids were found, of which one is assigned to NZSb11 [59,60]. All analyzed genomes were reconstructed from plant material, whereas GOE originated from insect tissue. In summary, the complete genome of the 16SrXII-P phytoplasma GOE represents the smallest genome of the stolbur phytoplasmas analyzed and is the first complete stolbur phytoplasma genome reconstructed from an insect host.

**Table 1:** Genomic features of strain GOE in comparison to the other complete 16SrXII phytoplasma genomes.

16SrXII-subgroup	16SrXII-P	16SrXII-A					16SrXII-B/-C	
Taxon		‘*Ca.* P. solani’					‘*Ca.* P. australiense’	
Strain	GOE	c1	c4	c5	o3		PAa	NZSb11
**Chromosome**								
Accession	CP155828	CP103788	CP103787	CP103786	CP103785		AM422018	CP002548
Length (bp)	704,525	751,320	751,188	824,084	973,640		879,324	959,779
GC content (%)	26.17	28.37	28.37	28.07	28.58		27	27
No. of CDSs (protein coding)	663	719	724	807	1000		684	1100
Coding density (genes/kb)	0.941	0.956	0.963	0.979	1.027		0.777	1.146
No. of rRNA operons	2	2	2	2	2		2	2
No. of tRNAs	32	32	32	32	32		35	35
No. of tmRNAs	1	1	1	1	1		1	1
No. of ncRNAs	2	2	2	2	2		2	2
**Plasmids**								
Accession								DQ318777
Length (bp)								3635
No. of CDSs (protein coding)								4
Host for reconstruction	*Pentastiridius* *leporinus*	Bindweed	Bindweed	Bindweed	Stinging nettle		Cotton	Strawberry
References	[35]	[61]					[62]	[59,63]

### 3.2. Phylogenetic Assessment

#### 3.2.1. Average Nucleotide Identity and Sequence Synteny

Since the actual guidelines for the taxonomic assessment of the genus ‘*Ca.* Phytoplasma’ suggest the use of average nucleotide identity (ANI) [64], we computed pairwise ANI for the analyzed stolbur phytoplasma chromosomes. ANIs for GOE compared to the other genomes ranged from 82 to 83% (Table 2). All other members of the taxon ‘*Ca.* P. solani’, including strains c1, c4, c5, and o3 showed identities higher than 98% when compared to each other, indicating species affiliation, while PAa and NZsb11 of the taxon ‘*Ca.* P. australiense’ ANIs ranged from 80% to 82%. Our ANI analysis is consistent with a recent sequence marker analysis that suggests that stolbur phytoplasmas of the 16SrXII-P subgroup should be considered as ‘*Ca.* P. solani’-related species [15]. Taken together, the ANI of GOE with a maximal value of 83% is clearly below 95%, which, according to the current taxonomic guidelines, indicates a new phytoplasma species [64].

**Figure 3:**
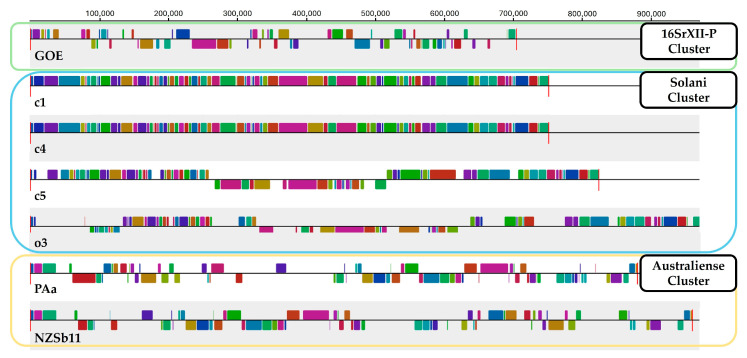
The sequence synteny of stolbur phytoplasma strains was determined using Mauve. Clusters obtained from ANI analysis were highlighted with green (16SrXII-P), blue (Solani), and yellow (Australiense) boxes surrounding the outer periphery. Sequence synteny is indicated by inner blocks of identical colors.

Furthermore, sequence synteny analysis with Mauve resulted in the formation of three clusters (Figure 3). Sequence synteny also showed the separation of strain GOE from the other clusters representative of the taxa ‘*Ca.* P. solani’ and ‘*Ca.* P. australiense’. Therefore, sequence synteny analysis supports cluster formation from ANI analysis.

#### 3.2.2. Marker Gene Analysis

BLAST analysis of the two complete 16S rRNA gene sequences showed that strain GOE shows 100% identity with the amplicon of the ‘*Ca.* P. solani’-related strain 916/22 of the 16SrXII-P subgroup [15]. ‘*Ca.* P. solani’ strains c1, c5, and o3 showed an identity of >99% and c4 (98.75%) to the reference strain STOL (AB639069) of the 16SrXII-A subgroup [64,65]. The complete 16S rRNA gene sequence of GOE shows 98.95% identity to STOL and ranges from 97.70% to 99.02% with o3 (99.02% and 98.89%), c1 (98.95%), c5 (98.95% and 98.89%), and c4 (97.70%). The identities for the ‘*Ca.* P. australiense’ strains range from 97.96% to 98.24% with PAa (98.24% and 98.05%) and NZSb11 (98.23 and 97.96%).

**Figure 4:**
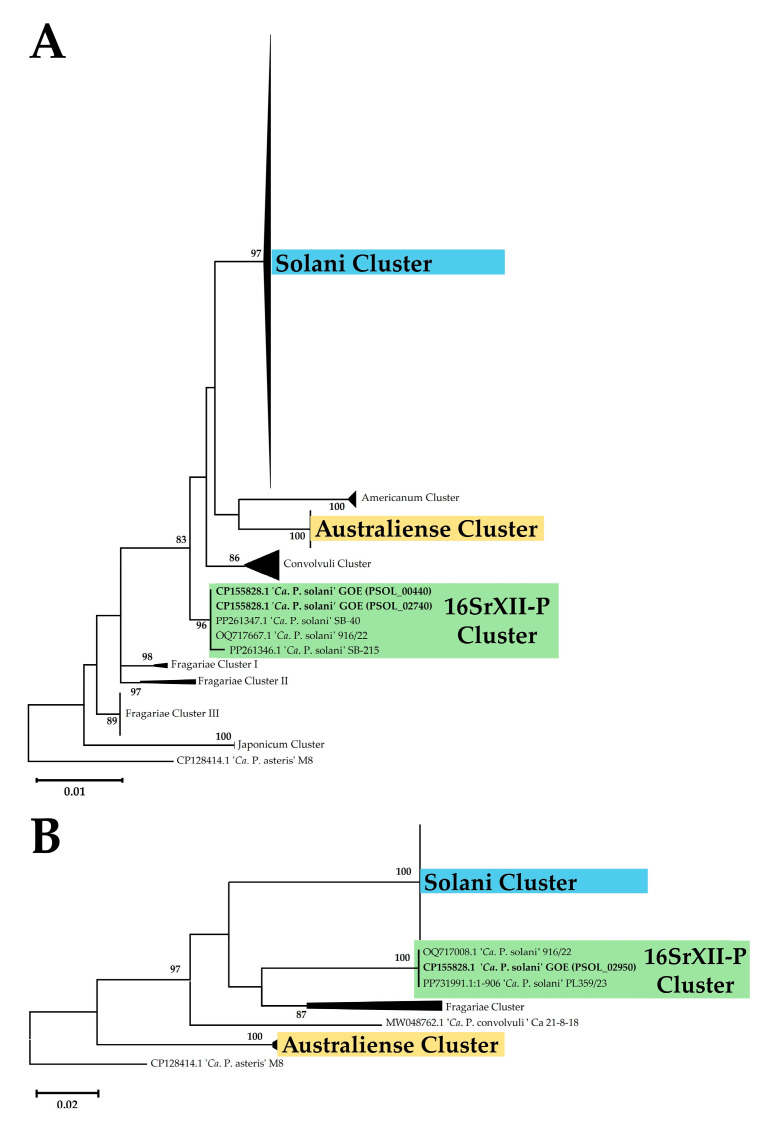
Maximum likelihood phylogeny based on the phytoplasma marker genes 16S rRNA (**A**) and *tuf* (**B**). Strain names with corresponding GenBank accessions are either given or listed in Table S1 in representative clusters. Only bootstrap support values of 70 or above are displayed, with data obtained from 1000 replicates. Scale bars indicate substitutions per site. Each cluster represents a distinct grouping of related sequences (Table S1). Clusters consistent with the whole-genome phylogeny are highlighted in the same color for clarity and comparison.

The maximum likelihood phylogeny of the R16F2n/R2 amplicon of the 16S rRNA showed the formation of nine clusters, whereby the strains of the analyzed genomes were divided into three clusters (Figure 4A). Strain GOE was assigned to a cluster with representative sequences for the 16SrXII-P subgroup. The strains c1, c4, c5, and o3 were assigned to a cluster mainly represented by sequences of ‘*Ca.* P. solani’, while the ‘*Ca.* P. australiense’ strains PAa and NZSb11 were assigned together (Table S1). Phylogenetic analysis of *tuf* analysis formed in total five clusters and confirmed the assignment of the analyzed strains to the same three clusters (Figure 4B). Additionally, the *tuf* analysis shows that GOE clusters besides other German strains also with the Polish 16SrXII-P strain PL359/23 (PP731991) [16].

**Figure 5:**
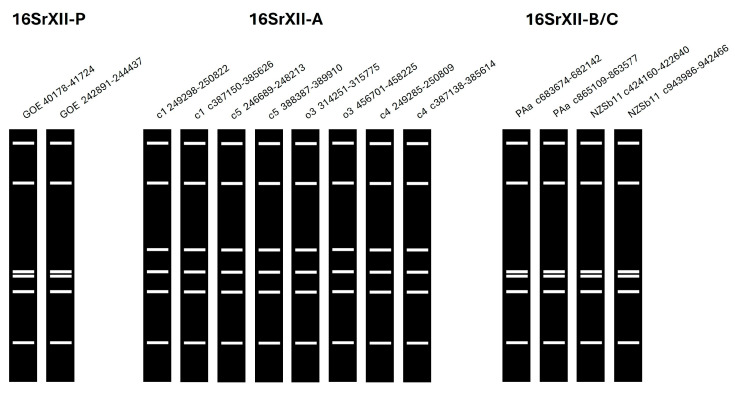
RFLP patterns from virtual gel images of 16S rRNA sequences from all analyzed stolbur genomes corresponding to the R16F2n/R2 amplicons obtained from in silico digestions with the key restriction enzyme MseI (Tru1I) in iPhyClassifier [45]. Numbers next to the strain names indicate the position in the genome and the letter “c” specifies a position on the reverse strand.

In silico RFLP analysis of the 16S rRNA of all stolbur genomes analyzed using the restriction enzyme MseI (Tru1I), which has been used to differentiate the 16SrXII-P subgroup [15], showed different patterns for the ‘*Ca.* P. solani’ 16SrXII-A subgroup but not for the ‘*Ca.* P. australiense’ 16SrXII-B/-C subgroups (Figure 5). The results of the virtual RFLP analysis for the analyzed stolbur strains across all 17 key restriction enzymes showed that GOE shared consistent patterns with the 16SrXII-P reference strain 916/22 (Figure S1A). RFLP patterns showed also that strains c1, c5, and o3 belong to 16SrXII-A with an 100% identity to the reference strain STOL (Figure S1B). Strain c4 differed by an identity of 97% and showed a different RFLP pattern by the key enzyme HinfI, indicating a different subgroup (Figure S1B). However, as BLAST analysis using the full-length 16S rRNA showed that strain c4 had the closest relationship to the 16SrXII-A subgroup strains c1, c5, and STOL, c4 appears to be a 16SrXII-A stolbur phytoplasma close to the subgroup boundary. The 16S rRNA sequences of PAa and NZSb11 showed interoperon heterogeneity when analyzed with the key enzyme BfaI, with one copy assigned to the 16SrXII-B and 16SrXII-C subgroups, respectively (Figure S1C) [66,67]. The key enzymes BfaI and HaeIII were identified to distinguish the 16SrXII-P phytoplasmas from the 16SrXII-B/-C subgroups by RFLP patterns. Taken together, RFLP analysis reaffirmed cluster assignment from whole-genome and marker-gene phylogeny.

The phylogenetic analyses on the whole-genome and single-gene level confirm the separation of 16SrXII-P phytoplasmas from other stolbur group members and underline the heterogeneity of phytoplasmas within the 16SrXII stolbur group.

### 3.3. Functional Comparison and Reconstruction

#### 3.3.1. Pan-Genome Analysis

**Figure 6:**
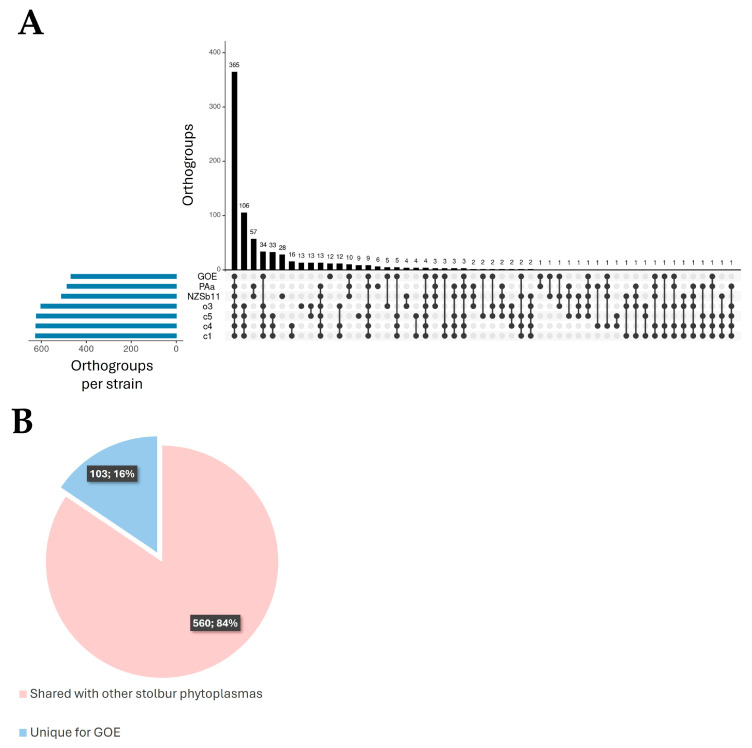
(**A**) Upset plot of predicted orthogroups. Black bars show the orthogroup number per intersection of respective strains (connected dots). The blue bar chart indicates the number of orthogroups per strain. (**B**) Pie chart indicating numbers of shared (pale red) and unique deduced protein sequences (blue) of strain GOE.

Orthogroup prediction of the analyzed stolbur phytoplasmas was performed with a data set of 5697 deduced amino acid sequences. Out of these, 5314 (93%) deduced amino acid sequences were assigned to 805 orthogroups. Overall, 365 orthogroups were predicted that were shared by all genomes analyzed (Figure 6A). This core set comprises 411 (~62%) deduced amino acid sequences of GOE that were assigned to proteins involved in functions that contribute to the maintenance of the phytoplasma cell. In total, GOE shares 560 (84%) of its deduced amino acid sequences, whereas 103 (16%) are unique and represent accessory components of the pan-genome including both species-specific and unassigned sequences (Figure 6B). This unique part comprises 100 deduced amino acid sequences (97%), which were annotated as hypothetical proteins, with five of these being secreted and 31 associated with the cytoplasmic membrane (Table S2). Therefore, GOE differs with features associated with pathogen–host interaction from other stolbur phytoplasmas.

#### 3.3.2. Metabolic Pathways

**Figure 7:**
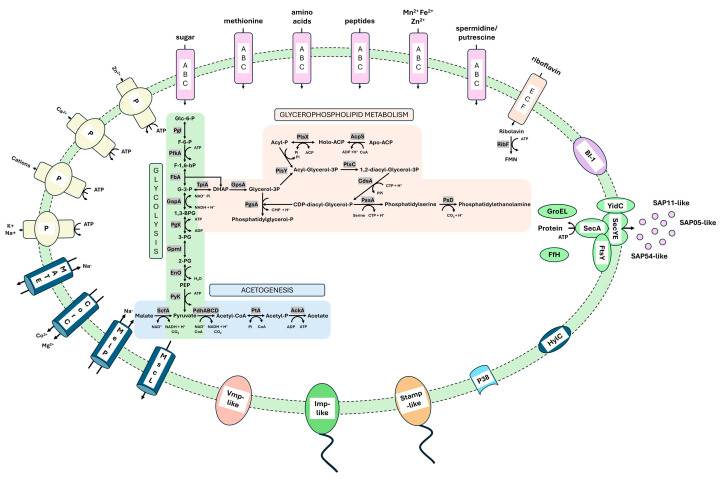
Schematic of complete metabolic pathways and representative membrane proteins involved in metabolism and virulence of the 16SrXII-P phytoplasma strain GOE.

The functional reconstruction showed that the chromosome of strain GOE encodes the reduced metabolism typical for phytoplasmas [32,33], including three complete metabolic modules (Figure 7), which were shared by all analyzed stolbur genomes. Two of these are involved in carbohydrate metabolism, namely the core module of glycolysis/glucogenesis and acetogenesis. Glucose-6-phosphate can be catabolized to pyruvate, followed by oxidation and acetogenesis. In addition, the conserved malate–acetate pathway of phytoplasmas starting from the oxidative decarboxylation of malate to pyruvate is encoded [33,68]. In addition to carbohydrate metabolism, the glycolysis core module is also linked to glycerophospholipid metabolism via phosphatidylethanolamine biosynthesis, which is thought to contribute to the maintenance of the phytoplasma cell membrane [33]. Interestingly, all the stolbur phytoplasma genomes analyed so far possess the gene *ribF* which encodes a riboflavin kinase that is suggested to be involved in FMN/FAD synthesis. FMN/FAD are important cofactors for enzymes involved in redox reactions. Only a few studies have reported the presence of a putative *ribF* in phytoplasmas outside the 16SrXII stolbur group [33]. Our analysis of GOE’s *ribF* revealed that members of the 16SrXIII group (Mexican periwinkle virescence group) also harbor a riboflavin kinase besides the stolbur group (Figure S2). Therefore, our analysis suggests that riboflavin kinase is a metabolic feature indicating a residual feature of the reductive evolution of phytoplasmas.

#### 3.3.3. Transporter

Due to their reduced metabolism, phytoplasmas rely on the exchange of essential metabolites from their host environment via several transporters [33]. A total of 34 genes encoding functional subunits of ATP-binding cassette (ABC) transporters were identified in GOE, including six genes of the energy coupling factor (ECF) transporter family involved in the uptake of amino acids, peptides, bivalent cations, polyamides, sugars, and riboflavin (Figure 7).

Besides the ABC transporters, all analyzed stolbur phytoplasma genomes encode four P-type ATPases for the export of zinc, calcium, sodium, potassium, and unselective cation transport. In addition, all genomes encode two multidrug and toxic compound extrusion (MATE) transporters, a cobalt and magnesium exporter CorC, and the large conductance mechanosensitive channel, as well as a malate/sodium symporter (MelP).

#### 3.3.4. Membrane-Associated Interaction

Phytoplasmas encode so-called immunodominant membrane proteins (IDPs) which are critical for their successful transmission and colonization of plant and insect hosts. Three main types of phytoplasma IDPs have been described: AMP, IMP, and IdPA [69]. Phytoplasmas of the 16SrXII stolbur group encode an AMP-type protein called stolbur antigenic membrane protein (STAMP), which is suggested to interact with actin filaments of their insect vectors [70]. Further, IMP is known for the interaction of phytoplasmas with plant actin [71]. All analyzed stolbur group members encode *stamp*, flanked by the genes *groES*, *groEL*, and *nadE*. *Imp* was also found to be ubiquitous in the analyzed stolbur genomes, flanked by the genes *rnc*, *dnaD*, and *pyrG*. Our analysis therefore supports that stolbur phytoplasmas share the common conserved genomic organization of phytoplasma IDPs as described previously [72].

**Figure 8:**
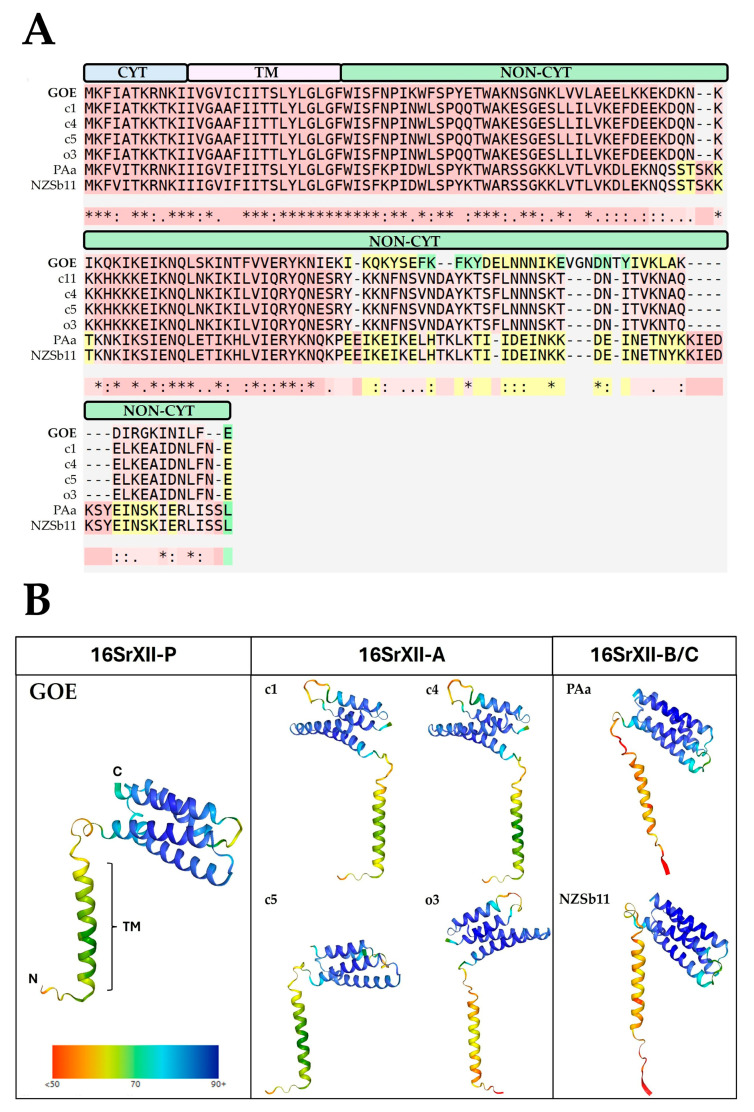
(**A**) Functional alignment of Imp using T-Coffee highlighting amino acid sequence conservation and chemical similarity. The peptide domain localization is shown above the alignment: cytoplasmic CYT (blue), transmembrane TM (pale pink), and non-cytoplasmic NON-CYT (green). Color code for amino acid conservation: Highly conserved (red), conserved but slightly variable (pale red), less conserved (yellow), and most variable (green). Asterisks (*) denote fully conserved residues, colons (:) represent strong chemical similarity, dots (.) indicate moderate similarity, and spaces show low or no conservation. (**B**) Structure prediction of Imp via AlphaFold. N and C indicate the N- and C-terminus of the protein, respectively. The colored bar indicates the percentage of prediction probability of the deduced protein structures.

As GOE was reconstructed from the insect vector *P*. *leporinus*, further in silico analysis of its deduced IMP-like protein sequence (PSOL_03490) was carried out for functionality and structure to investigate its suitability for transfer into the plant host (Figure 8).

The functional alignment of the IMP amino acid sequences revealed that the IMP-like protein of GOE shows conservation starting from the N-terminus (Figure 8A). All analyzed sequences possess a cytoplasmic, a transmembrane, and a non-cytoplasmic domain. The latter is involved in the interaction with the plant host and shows the highest sequence variation. In addition, all the sequences analyzed showed a similar tertiary structure (Figure 8B). Furthermore, the maximum likelihood phylogeny of Imp showed on nucleotide and amino acid levels the same clustering as described for the marker genes (Figure S3). These results indicate that IMP-like proteins of stolbur phytoplasmas differ mainly in their non-cytoplasmic interaction domain present in the host environment, which may be explained by their adaptation to different host plants and insect vectors.

The strain GOE encodes a gene for a protein similar to the adhesin P38 (PSOL_06560), which was conserved in all other compared stolbur phytoplasmas. P38 was initially described in onion yellows phytoplasmas and has been demonstrated to interact with both insect and plant hosts [73]. Within all analyzed genomes *p38* is flanked by *pyk*, encoding pyruvate kinase, and *folA*, which encodes a dihydrofolate reductase.

Further, GOE encodes a similar protein to the variable membrane protein 1 (PSOL_05990). Variable membrane proteins are highly divergent and are, like IDPs, suggested to be involved in the pathogen–host interactions of phytoplasmas [74]. All analyzed stolbur genomes encode Vmp1. In GOE, *vmp1* is flanked by the genes *uvrA*, encoding an exinuclease ABC subunit A, and *ribF*, encoding riboflavin kinase, while in c1, c4, c5, and o3, *vmp1* is flanked by *uvrA* and *ligA*, encoding a DNA ligase. The Vmp1 gene of strains PAa and NZSb11 is flanked by *ribF* and *engC*, which encodes a GTPase involved in the assistance of the maturation of ribosomes.

Another potential virulence factor identified in the membrane of strain GOE is the bax inhibitor 1 (BI-1) (PSOL_2960). BI-1 proteins are involved in the suppression of programmed cell death [75], but the exact mechanism of phytoplasma BI-1 is unknown. All analyzed stolbur genomes encode a gene for BI-1 flanked by the genes *tuf* and *rsmG*. This is consistent with other studies that have analyzed the coding sequence of BI-1 in phytoplasma genomes and support the hypothesis that BI-1 is an evolutionarily conserved virulence factor that adapts to the host environment, also for stolbur phytoplasmas [32,76,77].

Moreover, all analyzed stolbur phytoplasmas encode *hylC*, which encodes a hemolysin III-like protein that is reported as a conserved candidate virulence factor and marker in phytoplasmas [78]. The gene *hylC* is flanked by *ackA* coding for an acetate kinase within all genomes analyzed. In GOE, besides *ackA*, the gene *nfnB* encoding a nitro reductase is flanking *hylC* while in c1, c4, c5, and o3, *hylC* is also flanked by *hisS* encoding a histidine tRNA ligase in all analyzed stolbur phytoplasmas. PAa and NZsb11 are additionally flanked by *mscL* encoding the large conductance mechanosensitive channel.

Taken together, the genome of GOE and those of the analyzed members of the stolbur group encode most of the previously described membrane-associated, conserved virulence factors that have been described or suggested to be involved in the interaction of phytoplasmas with their hosts.

#### 3.3.5. Secretome Analysis

Secretome reconstruction showed that all analyzed stolbur phytoplasmas encode the common set of *sec* genes including *secA*, *secE*, *secY*, and *yidC* involved in the Sec-dependent secretion pathway representing the sole described secretion system in phytoplasmas yet [79]. In addition, of all analyzed genomes of strain GOE, the ‘*Ca.* P. solani’ strains c1, c4, c5, and o3 encode the complete gene set (*ffs*, *ffh*, *ftsY*) for the signal recognition particle (SRP) pathway. The SRP pathway drives the co-translational integration of membrane proteins by targeting ribosomes to the Sec-dependent membrane complex of the secretion pathway [80]. In contrast, only PAa encodes a functional Ffh within the analyzed ‘*Ca.* P. australiense’ genomes, while in NZSb11 *ffh* is disrupted, and it remains unclear whether an alternative protein fulfils the role of Ffh, as in the case of GroEL, which is considered to substitute for the function of the chaperone SecB in the Sec-dependent secretion system [33]. However, as little is known about functional redundancies within the SRP and Sec-dependent secretion pathways, this may indicate functional differences within the stolbur group in terms of protein translocation.

**Figure 9:**
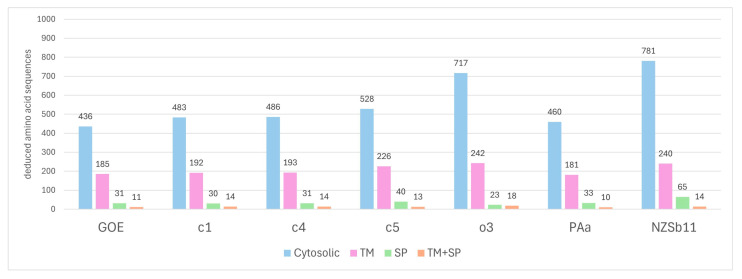
Number of deduced amino acid sequences that have been identified as either encoding a signal peptide (SP), transmembrane domain(s) (TM), or both (SP+TM) per strain.

In total, 196 (~30%) of the deduced amino acid sequences of strain GOE possess at least one transmembrane (TM) domain, of which eleven additionally harbor a signal peptide domain (SP+TM), whereas 31 (~5%) proteins were identified that have only a signal peptide domain (SP) representing putative effector proteins (Figure 9). The comparison of these results with those of the other analyzed stolbur phytoplasma genomes revealed that GOE harbors the lowest number of deduced amino acid sequences that are either present in the phytoplasma membrane or secreted into the host environment. In contrast, strain o3 displays the highest number of deduced amino acid sequences. Therefore, these results may indicate that with increasing genome size, the number of proteins associated with pathogen–host interaction in stolbur phytoplasmas is also increasing. This is following a previous genome study of phytoplasmas of the 16SrI asteris group [32].

#### 3.3.6. Important Effector Proteins

We identified proteins similar to the experimentally verified effectors of the secreted aster yellows phytoplasma witches’ broom proteins (SAPs) SAP11 (PSOL_01490) [81,82,83], SAP54 (PSOL_01470) [84], and SAP05 (PSOL_01730) [85] from ‘*Ca.* P. asteris’ strains. Strains c1 and c4 encoded also all three SAP family effectors analyzed, while the annotations of strains c5 and o3 describe SAP54 only. No homologs for the three analyzed SAPs were identified within the ‘*Ca.* P. australiense’ strains PAa and NZSb11 as described previously [62,86]. In comparison, the SAP11-like protein of GOE is enlarged in its C-terminal region compared to the identical SAP11-like proteins of c1 and c4 (111 aa) and shows an identity of 78.12%. In contrast to the SAP11-like protein, the SAP54-like protein (116 aa) of GOE showed a high sequence similarity with an identity of 89.74% to the identical SAP54-like proteins of strains c1 and c4, respectively, and 89.66% to the SAP54-like protein of strain c5 (116 aa), whereas the SAP54-like protein of o3 (97 aa) showed only a 39.78% identity. For SAP05, the homologs of strains GOE, c4, and c1 showed the same length of 322 amino acids and shared a sequence identity of 99.07%. Overall, the genome of strain GOE encodes all the well-characterized phytoplasma effectors of the SAP family, which have been described in many phytoplasmas and are located on transposable elements [81]. Furthermore, our analysis confirms the findings of a previous study that the coding of the SAP family effectors varies within the stolbur group [86].

**Figure 10:**
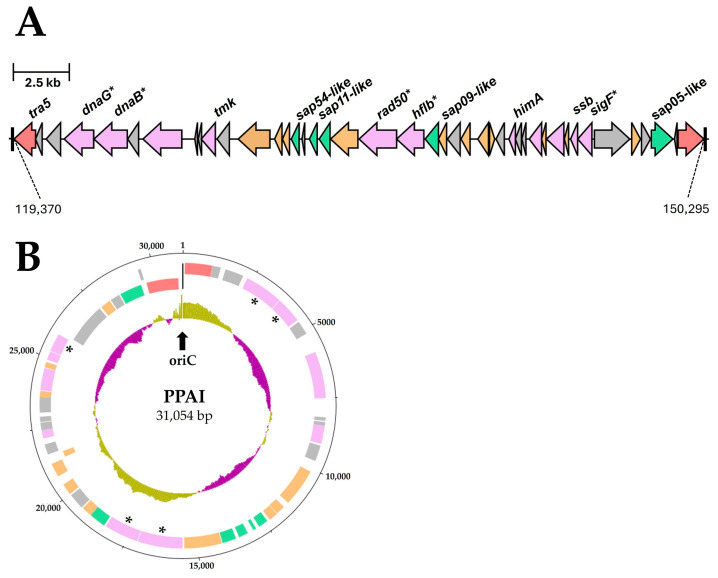
Phytoplasma pathogenicity island (PPAI) within the GOE chromosome (**A**) and its proposed extrachromosomal circular form (**B**). In the linear form (**A**), left-pointing arrows on the reverse strand and right-pointing arrows on the forward strand indicate coding orientation. Numbers connected by dashed lines indicate their position within the GOE genome. In the circular form (**B**), the following patterns are displayed from the outside in (1) outer ring representing the scale in base pairs, (2) predicted protein-coding sequences on the forward strand, (3) predicted protein-coding sequences on the reverse strand, and (4) GC skew analysis, where olive indicates above-average GC skew and violet indicates below-average GC skew. Genes are color-coded as follows: red—IS3 family transposases, purple—PMU-associated genes, orange—putative membrane proteins, green—putative secreted proteins. Asterisks indicate pseudogenes. Repeat regions are indicated by black bars. The black arrow marks the proposed *oriC* region.

Interestingly, all three SAP-like proteins analyzed are located on a transposon with a length of approximately 31 kb (Figure 10A), while for the ‘*Ca.* P. solani’ strains c1, c4, c5, and o3 the annotated SAP effectors are separately encoded on different locations in the chromosome but were also flanked by potential mobile unit (PMU)-associated genes. The complete transposon of GOE has an IS3 family at each end. Each IS element is represented by the gene *tra5*, which encodes a transposase flanked by an inverted repeat of 11 bp and a direct repeat of 3 bp per site (5′-(ACA)TTTTAAAAAnnnnnnTTTTTTTAAAA(ACA)-3′), located on the reverse strand, but which are not unique within the GOE chromosome sequence. These repeat regions have the typical size for IS elements but are shorter than those described for PMUs in the genomes of ‘*Ca.* P. asteris’ AYWB or ‘*Ca.* P. solani’ SA-1 [81,86,87]. The transposon harbors genes that are associated with PMUs and also replication such as *dnaG*, *dnaB*, *tmk*, *rad50*, *himA*, *ssb*, as well as *sigF* [81]. In addition, the transposon showed a similar GC content (27.85%) to the GOE chromosome overall with 26.17%. In silico circulation of the transposon suggests the formation of replicative intermediates indicated by a putative origin of replication (Figure 10B). This had been described for an extrachromosomal PMU-associated replicon of ‘*Candidatus* Phytoplasma asteris’ [88]. Taking into account the virulence factors encoded on the transposon, it must be considered as a complete phytoplasma pathogenicity island (PPAI).

## 4. Discussion

The 16SXII-P subgroup dominates the current phytoplasma infection in sugar beet in Germany and has also been detected in Poland [15,16]. However, the *P*. *leporinus*-driven epidemic is still ongoing [7,14,15,31] and it remains unclear to what extent the 16SrXII-P phytoplasma will spread throughout Germany and into other neighboring countries.

The comparative analysis of the 16SrXII-P subgroup from *P*. *leporinus* [35] highlighted the separated phylogenetic position and genomic content. This is in accordance with the first description of the 16SrXII-P phytoplasma 916/22 highlighting the distance to ‘*Ca.* P. solani’ but new species description was hampered by access to a limited number of genes and due to an 16S rDNA identity of 98.67% to the STOL reference [15], thereby missing the taxon threshold of 98.65% identity [64]. This also applies to the two complete and identical 16S rRNA genes of GOE showing 98.95% identity. Other gene markers can be used in addition with assigned thresholds comprising *tuf* (97.5%), *secY* (95%), *secA* (97.5), *rplV*-*rpsC* (97.5%), or *groEL* (97.6%) [64] and had been clearly fulfilled by amplicon sequence analysis of *tuf*, *secY*, and *rp*-locus for 916/22 standing in opposite to the 16S rRNA result [15]. However, an ANI value of <95% is considered as overvoting the criterium, if 16S rDNA identity is >98.65% [64]. While no genome of the reference strain STOL is available, a maximal ANI of 83.04% (strain c1) is reached in the analyzed complete genomes (Table 2). In contrast, ANI values of c1, c4, c5, and o3 range from 99.99% (c1, c4) to a minimum of 98.42% in ‘*Ca.* P. solani’, indicating one taxon for this criterium. Separation of the 16SrXII-P subgroup is also obvious in comparison to ‘*Ca.* P. australiense’, reaching a maximum of 82.23%. It is also notable that the ‘*Ca.* P. solani’ strains fulfil taxon separation from GOE (max. 83.05%) and ‘*Ca.* P. australiense’ (max. 80.12%).

Our functional reconstruction and comparison showed that the 16SrXII-P phytoplasma GOE and the other stolbur phytoplasmas analyzed encode the minimal metabolism with the core module of glycolysis coupled with acetogenesis as the energy-yielding instance as well as the glycerophospholipid metabolism for the preservation of the cytoplasmic membrane, as described previously for the genomes of the 16SrI asteris group [32]. This minimal coding of metabolic pathways can still differ between phytoplasma groups, as for example the 16SrX group member ‘*Ca.* P. mali’ lacks the energy-producing part of glycolysis from D-glyceraldehyde-3-phosphate to pyruvate [33,89], while phytoplasmas of the 16SrV group encode the carboxylic acid metabolism from malate to lactate [90]. However, neither of these features could be confirmed for the analyzed genomes of the stolbur group. What is noteworthy is that our analysis revealed the coding of a riboflavin kinase, which has been suggested to be involved in FMN/FAD synthesis as an important co-factor and appears to be a lost pathway outside of the 16SrXII stolbur group and the closely related 16SrXIII Mexican periwinkle virescence group. Apart from the synthesis of FMN/FAD, no information is available on the role of riboflavin in phytoplasmas in the context of uptake from the host environment. It has been shown that riboflavin levels are linked to the induction of plant resistance against phytopathogenic bacteria such as *Pseudomonas syringae* pv. tomato in *Arabidopsis thaliana* [91]. One may speculate that the uptake and utilization of riboflavin is involved in the immunomodulation of the host’s defense system. This would then be another brick in the series of host manipulations. As phytoplasmas are limited to the plant phloem, they secrete effector proteins to manipulate their hosts via the Sec-dependent secretion pathway as the major functional secretion system of phytoplasmas [79]. The secreted aster yellows witches’ broom proteins (SAPs) [81] represent with their well-examined effectors SAP11 [82], SAP54 [84], and SAP05 [85] an important group of virulence factors of phytoplasmas that cause growth abnormalities and interfere with the host hormone system [92]. We identified homologs of the phytoplasma effectors SAP11, SAP54, and SAP05 within the genome sequence of GOE, c1, and c4, whereas for c5 and o3 only a homolog for SAP54 was identified, and for the ‘*Ca.* P. australiense’ strains PAa and NZSb11, no homologs have been identified. Such a variable coding of these effectors has been shown also in other phytoplasma groups like the 16SrI asteris group [32,93]. It has been demonstrated that the SAP11 homolog of ‘*Ca.* P. solani’ alters plant morphology by destabilization of TEOSINTE BRANCHED 1-CYCLOIDEA-PROLIFERATING CELL FACTOR (TCP) transcription factors (TFs) when expressed in *A. thaliana.* Plants showed reduced biomass, size, proliferation, crinkled leaves, as well as deformations in the root system [83]. Further, SAP05 is known to induce massive proliferation in *A*. *thaliana* but with a different mechanism by degradation of the SQUAMOSA promoter binding protein-like genes and GATA motif-specific TFs independent from ubiquitination [85]. SAP54-like proteins degrade MADs-box transcriptional factors leading to the development of leaf-like flowers and therefore sterile plants [84]. No information by the infection on the seed production is available to date. Besides phytoplasma host plants, SAP11 and SAP54 are also suggested to have an influence on the behavior of the insect vectors of phytoplasmas in terms of attractiveness and reproduction [82,94,95], which needs clarification for the *P*. *leporinus* vector system. The presence of crucial phytoplasma effectors fits the observed symptoms associated to infection by ‘*Ca.* A. phytopathogenicus’ and/or 16SrXII-P phytoplasma in sugar beet in the field. Nevertheless, the exact mode of action of the SAP family proteins and how they interact with the transcription factors of affected plants and insect vectors remains elusive.

The analysis of the coding of effector proteins in phytoplasma genomes revealed their location on regions that are flanked by *tra5* genes of the IS3 family that form putative transposable elements which were named potential mobile units (PMUs) that are present as multiple fragmented copies in the phytoplasma genome [81]. They were also suggested to have the potential to excise from the chromosome, as they could also be detected as extrachromosomal replicons in high copy numbers in the host plant and insect [88]. Comparative analyses of PMUs across different phytoplasma species led to the hypothesis that PMUs can be exchanged by horizontal gene transfer and that effector proteins evolve faster than other genes not located on a PMU, explaining the species- or strain-specific encoding of effector proteins and leading to lineage-specific adaptation to different hosts [96,97,98]. We identified to our knowledge the first complete transposon harboring orthologs of the effector proteins SAP11, SAP54, and SAP05, representing a transposable phytoplasma pathogenicity island (PPAI) with a size of ~31 kb, suggesting the ability to excise as a replicon (Figure 10). In contrast, the circular PMU1 of the ‘*Ca.* P. asteris‘ strain AY-WB is with a total size of ~19 kb shorter and lacks the coding of homologs to the well-examined SAPs. Furthermore, in contrast to GOE, the inverted repeats of PMU1 from AY-WB show a size of 327–328 bp, which is atypical for IS3 family transposases, but both indicate an *oriC* in these regions when GC skew was analyzed [88]. Considering that this transposon has a similar GC content to the entire GOE chromosome, this suggests that such transposons entered phytoplasmas early on and evolved with them. It should also be considered that the transposon PPAI may be frequently available in the phloem of the host plant or in the insect vector as demonstrated for PMU1 [88] and can be taken up horizontally from other phytoplasmas and bacteria in the phloem or as suggested for other phytoplasmas [97]. It has been described that selfish DNA in prokaryotes can be integrated into eukaryotic plant genomes like IS elements [99]. It is therefore possible to speculate whether the transposon reported in the GOE genome is still functional and can be also integrated into the plant host. If so, such pathogenicity islands could explain the effective host adaptation of phytoplasmas, as long-term integration of effectors into and expression by the host could have led to an effective colonization strategy that overcomes the plant defence system. Furthermore, such a host-mediated effector expression would be of great breeding importance for phytoplasma-infected crops since the development of symptoms triggered by the effector expression independent from an infection would bias the selection of tolerant varieties.

## 5. Conclusions

This comparative genomic study of phytoplasma strain GOE provides deep insights into a representative of the important sugar beet stolbur pathogen subgroup 16SrXII-P, which is transmitted by the cixiid vector *P*. *leporinus* causing the current epidemic in Germany. Phylogenetic genome and single-gene analyses underline the differentiation from other stolbur taxa, while genomic benchmarks and functional reconstruction emphasize the general reductive evolution of phytoplasmas as highly adapted bacteria for vector and host infection. This includes the discovery of a complete pathogenicity island formed by a transposon containing the major effector proteins of phytoplasmas, providing new insights into evolutionary mechanisms and a starting point for further studies to analyze spread, but also the manipulation of organogenesis in sugar beet by encoded effectors.

## Figures and Tables

**Table 2:** Average nucleotide identities of stolbur phytoplasma genome sequences.

Cluster	16SrXII-P	Solani				Australiense	
Strain	GOE	c1	c4	c5	o3	PAa	NZSb11
GOE	-	**83.04**	**83.05**	**82.87**	**82.85**	**82.00**	**82.23**
c1	**83.04**	-	99.99	99.15	98.42	**79.71**	**79.99**
c4	**83.05**	99.98	-	99.15	98.40	**79.73**	**80.04**
c5	**82.87**	99.15	99.15	-	98.53	**79.94**	**80.02**
o3	**82.85**	98.42	98.40	98.53	-	**80.12**	**79.57**
PAa	**82.00**	**79.71**	**79.73**	**79.94**	**80.12**	-	98.64
NZSb11	**82.23**	**79.99**	**80.04**	**80.02**	**79.57**	98.64	-

Bold values indicate crossing the species affiliation threshold of 95 percent [64]. ANI clusters are highlighted as follows: 16SrXII-P (green), Solani (blue), and Australiense (yellow).

## Data Availability

The datasets generated during and/or analyzed during the current study can be find in the main text and the Supplementary Materials.

## Supplementary Materials

The following supporting information can be downloaded at https://www.mdpi.com/article/10.3390/pathogens14020180/s1, Figure S1: Virtual gel images of the RFLP analysis of the analysed stolbur genomes and representative reference strains obtained from the digestion with all 17 key restriction enzymes. The gels are grouped according to their 16SrXII subgroup affiliation as follows: (A) 16SrXII-P, (B) 16SrXII-A, and (C) 16SrXII-B/-C. Corresponding accessions and positions in the genome sequences are indicated at the bottom of each virtual gel image; Figure S2: Maximum likelihood phylogeny based on the gene *ribF*. Names of strains with their respective GenBank accession are given. 16Sr group/subgroup affiliation is highlighted next to the clusters. Only bootstrap support values of 70 or above are displayed, with data obtained from 1,000 replicates. Scale bars indicate substitutions per site; Figure S3: Maximum likelihood phylogeny of the analysed stolbur genomes based on the gene *imp* (A) and the deduced amino acid sequences of Imp (B). Names of strains with their respective GenBank accession are given. Only bootstrap support values of 70 or above are displayed, with data obtained from 1,000 replicates. Scale bars indicate substitutions per site; Table S1: Cluster composition of the maximum likelihood phylogeny; Table S2: Unique deduced amino acid sequences of 16SrXII-P phytoplasma strain GOE.
