# Supplementary material for: The 16SrXII-P Phytoplasma GOE Is Separated from Other Stolbur Phytoplasmas by Key Genomic Features
Source: Pathogens. 2025 Feb 11;14(2):180. doi: 10.3390/pathogens14020180 (PMC11857868; doi:10.3390/pathogens14020180)
Supplement: Supplementary file 1 [file pathogens-14-00180-s001.zip › pathogens-3452146-supplementary.pdf]

Table S1. Cluster composition of the maximum likelihood phylogeny.

Marker	16S rRNA		*tuf*	
Cluster	Source name	Accession	Source name	Accession
Solani Cluster	'*Ca.* P. solani' clone Aragimahale1	MT627355.1	**'*Ca.* P. solani' c1 (psc1_03600)**	**CP103788.1**
	'*Ca.* P. solani' clone Aragimahale5	MT627357.1	**'*Ca.* P. solani' c5 (psc5_04100)**	**CP103786.1**
	'*Ca.* P. solani' clone Aragimahale3	MT627356.1	**'*Ca.* P. solani' c4 (psc4_03650)**	**CP103787.1**
	Dioscorea communis phytoplasma clone PM5	MF002596.1	Stolbur phytoplasma draft strain 284/09	FO393427.1
	'*Ca.* P. solani' isolate Van 3	KY579358.1	Phytoplasma sp. BN-Op37	GU220562.1
	'*Ca.* P. solani' clone Tut 78	OK336713.1	'*Ca.* P. solani' isolate 3-21-36	EU814646.1
	'*Ca.* P. solani' clone Tut 7	OK336714.1	'*Ca.* P. solani' isolate 1-38-40	EU552455.1
	'*Ca.* P. solani' clone Tut 20	OK336717.1	'*Ca.* P. solani' isolate 25-17-14	EU814638.1
	'*Ca.* P. solani' isolate PE147	OR295221.1	'*Ca.* P. solani' isolate 25-5-48	EU814641.1
	'*Ca.* P. solani' isolate PP164	OR736053.1	'*Ca.* P. solani' isolate RU106/23	PP731983.1
	'*Ca.* P. solani' clone Tut 3	OK336715.1	'*Ca.* P. solani' isolate FR665/23	PP731989.1
	'*Ca.* P. solani' clone Tut 2	OK336716.1	'*Ca.* P. solani' isolate R47/5	FJ394552.1
	'*Ca.* P. solani' strain VAN1	KJ957010.1	Phytoplasma sp. STOL11	JQ797670.1
	'*Ca.* P. solani' clone 2E2HF9	MH088869.1	'*Ca.* P. solani' isolate 26-16-33	EU814643.1
	Phytoplasma sp. BN-Ma193	FJ409897.1	'*Ca.* P. solani' isolate RU122/23	PP731985.1
	'*Ca.* P. solani' clone Tut 1	OK336718.1	'*Ca.* P. solani' isolate AZ GR23-14	LT899728.1
	'*Ca.* P. solani' strain VAN1 clone 2	KJ957011.1	'*Ca.* P. solani' isolate HYT2	KC243394.1
	**'*Ca.* P. solani' c1 (psc1_r00010)**	**CP103788.1**	'*Ca.* P. solani' isolate F11	DQ418459.1
	'*Ca.* P. solani' clone TT-27	OR095673.1	'*Ca.* P. solani' strain 429/19	MT157234.1
	'*Ca.* P. solani' clone Sgt1	PP837564.1	'*Ca.* P. solani' isolate FR662/23	PP731987.1
	'*Ca.* P. solani' strain 241/13	KF907506.1	'*Ca.* P. solani' isolate F9	DQ418456.1
	'*Ca.* P. solani' isolate Z187	OQ703963.1	'*Ca.* P. solani' isolate R1V	DQ418460.1
	'*Ca.* P. solani' isolate P1-1 clone P1-1	LR584984.1	'*Ca.* P. solani' isolate CC2	MZ970606.1
	'*Ca.* P. solani' isolate 415 05	EU010009.1	'*Ca.* P. solani' isolate F5	DQ418458.1
	'*Ca.* P. solani' clone 65-C	MF564267.1	'*Ca.* P. solani' isolate M1V	DQ418457.1
	**'*Ca.* P. solani' o3 (pso3_r00010)**	**CP103785.1**	'*Ca.* P. solani' isolate R49/15	FJ394551.1
	Phytoplasma sp. BN-Fc55	EU836653.1	Phytoplasma sp. BN-Fc6	GU220558.1
	'*Ca.* P. solani' isolate 85 04	EU014776.1	**'*Ca.* P. solani' o3 (pso3_05120)**	**CP103785.1**
	Phytoplasma sp. BN-Fc213	EU836644.1	'*Ca.* P. solani' strain PFY	KC481242.1
	'*Ca.* P. solani' strain BN-Ab151	KX010828.1	'*Ca.* P. solani' isolate TH-NKT-P26	MW464322.1
	'*Ca.* P. solani' isolate 2642BN	AJ964960.1	'*Ca.* P. solani' isolate TH5-5	MW464320.1
	Phytoplasma sp. BN-Ab150	FJ409891.1	'*Ca.* P. solani' isolate TH3-2	MW464318.1
	Iranian hemp witches-broom phytoplasma strain HAY4	JF441273.1	'*Ca.* P. solani' isolate TH-NKT015	MW464321.1
	Iranian hemp witches-broom phytoplasma strain HAY2	JF441274.1	'*Ca.* P. solani' isolate AZ GR05-15	LT899723.1
	'*Ca.* P. solani' isolate 51-3 clone 51	LR782095.1	'*Ca.* P. solani' isolate BN-DG23	MT505886.1
	Phytoplasma sp. BN-Ma192	FJ409896.1	'*Ca.* P. solani' isolate BN-a8	MT505885.1
	'*Ca.* P. solani' isolate G66	JN887313.1	'*Ca.* P. solani' isolate I8	MZ970607.1
	'*Ca.* P. solani' isolate B18	KF614623.1	'*Ca.* P. solani' partial isolate AZ GR21-14	LT899727.1
	Russia potato purple top phytoplasma Rus-PPT124	EU344890.1	'*Ca.* P. solani' isolate I10	MZ970609.1
	'*Ca.* P. solani' isolate VV1395	OL873119.1	'*Ca.* P. solani' isolate Tom46Mar1	ON864440.1
	'*Ca.* P. solani' isolate 3-21-36	EU814645.1		
	'*Ca.* P. solani' strain GE05	KF996536.1		
	Stolbur-Rus phytoplasma strain Rus93	GU004375.1		
	'*Ca.* P. solani' isolate 58-2 clone 58	LR782097.1		
	'*Ca.* P. solani' isolate 400 05	EU010007.1		
	'*Ca.* P. solani' strain 429/19	MT157232.1		
	'*Ca.* P. solani' clone 5043	JX311953.1		
	Convolvulus arvensis stolbur phytoplasma clone P1/P7-Conv2/2010-Bg	JN561702.1		
	'*Ca.* P. solani' clone BE16 16S	MF185362.1		
	'*Ca.* P. solani' isolate 26-16-33	EU814644.1		
	Russia potato purple top phytoplasma Rus-PPT111	EU344889.1		
	Stolbur-It phytoplasma strain PTV	GU004374.1		
	'*Ca.* P. solani' strain BN-Ab146	KX010827.1		
	'*Ca.* P. solani' clone SY3	MF184927.1		
	'*Ca.* P. solani' isolate 25-5-48	EU814640.1		
	Phytoplasma sp. BN-Op37	EU836656.1		
	Grapevine stolbur phytoplasma strain GrIRAN08	GQ403235.1		
	'*Ca.* P. solani' strain 284/09	JQ730740.1		
	'*Ca.* P. solani' strain 142/09	JQ730739.1		
	'*Ca.* P. solani' strain 224/09	JQ730742.1		
	'*Ca.* P. solani' isolate 104 04	EU014779.1		
	'*Ca.* P. solani' isolate ML NS-2016	MF503627.1		
	Russia potato purple top phytoplasma Rus-PPT94	EU344885.1		
	'*Ca.* P. solani' strain 121/09	JQ730750.1		
	**'*Ca.* P. solani' c1 (psc1_r00060)**	**CP103788.1**		
	Phytoplasma sp. BA	JQ868436.1		
	'*Ca.* P. solani' strain 198/10	JQ730743.1		
	'*Ca.* P. solani' clone TT-31	OR095682.1		
	'*Ca.* P. solani' isolate Ei22	MN047263.1		
	'*Ca.* P. solani' isolate FR662/23	PP716918.1		
	Corn-reddening phytoplasma 2005/2	DQ222972.1		
	'*Ca.* P. solani' isolate 20B	PP153620.1		
	'*Ca.* P. solani' isolate 8K	PP153622.1		
	'*Ca.* P. solani' isolate 27K	PP153621.1		
	'*Ca.* P. solani' strain Rus-907c13	KP864674.1		
	Russia potato purple top phytoplasma Rus-PPT92	EU344884.1		
	'*Ca.* P. solani' strain Rus-AWB803F	KY587524.1		
	'*Ca.* P. solani' strain Rus-AWB804F	KY587525.1		
	'*Ca.* P. solani' strain 204/10	JQ730744.1		
	'*Ca.* P. solani' strain 125/10	JQ730745.1		
	'*Ca.* P. solani' strain R13 72	KF583785.1		
	'*Ca.* P. solani' strain R13 69	KF583787.1		

	'*Ca.* P. solani' isolate 25-17-14	EU814637.1		
	Grapevine yellows phytoplasma Yazd isolate Chahgyre	KX355748.1		
	'*Ca.* P. solani' clone CAN 133	MZ477864.1		
	'*Ca.* P. solani' isolate Lorestan	KF130968.1		
	'*Ca.* P. solani' isolate Fars	KF130967.1		
	'*Ca.* P. solani' clone CAN 139	MZ477866.1		
	'*Ca.* P. solani' clone TEK 57	MZ477868.1		
	'*Ca.* P. solani' clone TEK 50	MZ477862.1		
	'*Ca.* P. solani' strain STOL	AB639070.1		
	Sugarcane phytoplasma strain Mauritius	AJ539181.1		
	Tomato big bud phytoplasma Kazeroon	KX098490.1		
	Stolbur phytoplasma strain STOL clone STOL11	AF248959.1		
	'*Ca.* P. solani' clone PW30	KY398726.1		
	'*Ca.* P. solani' clone Van 65	KT595210.1		
	Iranian alfalfa witches-broom phytoplasma isolate Mes 45	KT763372.1		
	Iranian alfalfa witches-broom phytoplasma isolate Mes 37	KT763371.1		
	Iranian alfalfa witches-broom phytoplasma isolate Mes 30	KT781662.1		
	Iranian alfalfa witches-broom phytoplasma isolate Mes 95	KT750060.1		
	'*Ca.* P. solani' isolate 93 04	EU014778.1		
	Phytoplasma sp. BN-Ma182	FJ409895.1		
	'*Ca.* P. solani' clone TUR Tom 432 HC	MT992796.1		
	'*Ca.* P. solani' clone TUR Bin 917 HC	MT993422.1		
	Russia potato purple top phytoplasma Rus-PPT109	EU344888.1		
	Phytoplasma sp. BN-Fc13	EU836649.1		
	Phytoplasma sp. BN-Op30	EU836652.1		
	Phytoplasma sp. BN-Ma198	FJ409898.1		
	Bois noir phytoplasma strain CH-1	HQ589193.1		
	Phytoplasma sp. BN-Ma202	FJ409899.1		
	'*Ca.* P. solani' clone Van D3	MF576263.1		
	'*Ca.* P. solani' isolate WB-16	OQ297265.1		
	'*Ca.* P. solani' clone 161/16	KY579338.1		
	'*Ca.* P. solani' isolate RU106/23	PP716917.1		
	'*Ca.* P. solani' isolate L1-10	KP324844.1		
	'*Ca.* P. solani' isolate PD230	OR736055.1		
	'*Ca.* P. solani' strain 138/10	JQ730746.1		
	'*Ca.* P. solani' partial 16S rRNA gene isolate 86-3	LR782096.1		
	Phytoplasma sp. BN-Op224	EU836658.1		
	'*Ca.* P. solani' isolate 77-4 clone 77	LR782094.1		
	'*Ca.* P. solani' strain STOL	AB639069.1		
	Paper flower yellows phytoplasma strain PFY	JX128698.1		
	Pumpkin witches-broom phytoplasma clone PuWB3	MH718397.1		
	Cucurbita moschata witches-broom phytoplasma isolate PuWB-XJ	MH731275.2		
	'*Ca.* P. solani' strain SX-CP clone SZ-8	KT844650.1		
	'*Ca.* P. solani' strain SX-CP clone LN-3	KT844645.1		
	'*Ca.* P. solani' Kamibun	LC460259.1		
	Papaya phytoplasma TW	AJ919994.2		
	Phytoplasma sp. strain 404	MN294449.1		
	'*Ca.* P. solani' strain SX-CP clone LN-2NA	KT844644.1		
	'*Ca.* P. solani' isolate TH7-2	MW464326.1		
	'*Ca.* P. solani' isolate TH-NKT-PK2	MW464327.1		
	'*Ca.* P. solani' strain SX-CP clone LY-4	KT844646.1		
	'*Ca.* P. solani' strain SX-CP clone LY-5	KT844647.1		
	Phytoplasma sp. strain 367	MN294438.1		
	Phytoplasma sp. strain 371	MN294441.1		
	'*Ca.* P. solani' isolate TH-NKT-PK9	MW464328.1		
	'*Ca.* P. solani' isolate TH11-20	MW464329.1		
	'*Ca.* P. solani' strain SX-CP clone LY-6	KT844648.1		
	'*Ca.* P. solani' strain SX-CP clone LN-1	KT844643.1		
	'*Ca.* P. solani' strain SX-CP clone SZ-9	KT844651.1		
	'*Ca.* P. solani' isolate LN-a	KT899861.1		
	'*Ca.* P. solani' strain SX-CP clone SZ-7	KT844649.1		
	Phytoplasma sp. strain 353	MN294437.1		
	Carica papaya phytoplasma clone NKT015	MW533147.1		
	'*Ca.* P. solani' isolate LN-b	KT899862.1		
	'*Ca.* P. solani' clone KRI-M-18	PQ333136.1		
	'*Ca.* P. solani' isolate 409 05	EU010008.1		
	'*Ca.* P. solani' isolate PorrocaC5	EU131021.1		
	Phytoplasma sp. BN-Op125	EU836646.1		
	Phytoplasma sp. BN-Fc3	EU836647.1		
	Phytoplasma sp. BN-Ab175	FJ409894.1		
	Shahrekord (Iran) cucumber phyllody phytoplasma clone Cus2	MK402983.1		
	Vitis sp. yellow leaf phytoplasma clone Farsejin	MN877919.1		
	Vitis sp. yellow leaf phytoplasma clone Ziaabad	MN877920.1		
	Capsicum sp. yellow leaf phytoplasma clone Alvand	MN877916.1		
	Iranian alfalfa witches-broom phytoplasma isolate Mes 35	KT763373.1		
	'*Ca.* P. solani' clone 2180 Chardonnay	OQ120464.1		
	'*Ca.* P. solani' clone Rus-1999F	OQ130742.1		
	'*Ca.* P. solani' clone 2175 Chardonnay	OQ120463.1		
	Turkish potato stolbur phytoplasma	HM485579.1		
	'*Ca.* P. solani' isolate dh3	HQ402915.1		
	Viburnum macrocephalum phytoplasma isolate DHHQ	KX257028.1		
	Artemisia scoparia witches-broom phytoplasma isolate ASWB	KT899994.1		
	Artemisia scoparia witches-broom phytoplasma	KU759570.1		
	'*Ca.* P. solani' isolate BN44948-F2nR2-c2 SP6 Plate 1 14	MH279537.1		
	Iranian alfalfa phytoplasma M21	JQ412100.1		
	'*Ca.* P. solani' isolate 65-1	KY579357.1		
	**388387-389910 '*Ca.* P. solani' strain c5 (psc5_r00010)**	**CP103786.1**		
	**246689-248213 '*Ca.* P. solani' strain c5 (psc5_r00040)**	**CP103786.1**		

	**'*Ca.* P. solani' strain c4 (psc4_r00010)**	**CP103787.1**		
	**'*Ca.* P. solani' strain c4 (psc4_r00060)**	**CP103787.1**		
	'*Ca.* P. solani' strain Sh1	KC835139.1		
	'*Ca.* P. solani' isolate 425 05	EU010010.1		
	'*Ca.* P. solani' isolate 391 05	EU010006.1		
	'*Ca.* P. solani' isolate dom	HQ402916.1		
	'*Ca.* P. solani' strain BN-Ma178	KX010829.1		
	**'*Ca.* P. solani' o3 (pso3_r00040)**	**CP103785.1**		
	Phytoplasma sp. BN-Fc24	EU836655.1		
	'*Ca.* P. solani' isolate P8	PP261349.1		
	'*Ca.* P. solani' isolate 92 04	EU014777.1		
	'*Ca.* P. solani' isolate 134 04	EU014780.1		
	'*Ca.* P. solani' isolate 06PS085	EU086529.1		
	Phytoplasma sp. BN-Fc11	EU836645.1		
	Phytoplasma sp. BN-Op261	EU836650.1		
	Phytoplasma sp. BN-Ab164	FJ409892.1		
	Phytoplasma sp. BN-Fc12	EU836648.1		
	Phytoplasma sp. BN-Op121	EU836651.1		
	Phytoplasma sp. BN-Ab170	FJ409893.1		
	Phytoplasma sp. BN-Fc89	EU836654.1		
	Phytoplasma sp. BN-Op123	EU836657.1		
Australiense Cluster	**'*Ca.* P. australiense' PAa (c683674-682142)**	**AM422018.1**	**'*Ca.* P. australiense' PAa (PA0660)**	**AM422018.1**
	Strawberry green petal phytoplasma	AJ243044.1	Strawberry lethal yellows phytoplasma (CPA) NZSb11	DQ096804.1
	**Strawberry lethal yellows phytoplasma (CPA) NZSb11 (SLY_1141)**	**CP002548.1**	**Strawberry lethal yellows phytoplasma (CPA) NZSb11 (SLY_0469)**	**CP002548.1**
	Strawberry lethal yellows phytoplasma	AJ243045.1		
	Mollicutes sp. associated with papaya dieback disease	Y10095.1		
	'*Ca.* P. australiense' strain NZ09156	FJ943262.1		
	Senna surattensis stem fasciation phytoplasma isolate STSF-YNa1	JN192454.1		
	'*Ca.* P. australiense'strain SA03	KY012740.1		
	**'*Ca.* P. australiense' PAa (c865109-863577)**	**AM422018.1**		
	**Strawberry lethal yellows phytoplasma (CPA) NZSb11 (SLY_1167)**	**CP002548.1**		
	Mycoplasma sp.	X95706.1		
	Strawberry virescence phytoplasma isolate 3101	AY377868.1		
	'*Ca.* P. australiense' strain SA01	KY012738.1		
	'*Ca.* P. australiense' strain SA02	KY012739.1		
	Phormium yellow leaf phytoplasma	U43569.1		
	Phormium yellow leaf phytoplasma	U43570.1		
Fragariae Cluster I	'*Ca.* P. fragariae'' strain YN-10G	EU293842.1	'*Ca.* P. fragariae' isolate YN-169	KJ144900.1
	'*Ca.* P. fragariae'' strain CN-10A	HQ599234.1	'*Ca.* P. fragariae' isolate YN-10G	KJ144897.1
			'*Ca.* P. fragariae' isolate 165yn	KJ144899.1
Fragariae Cluster II	'*Ca.* P. fragariae'' strain YN-16	EU338445.1		
	'*Ca.* P.' sp. isolate BOMWB-YNym	OP642368.1		
Fragariae Cluster III	'*Ca.* P. fragariae' clone D598 18	MK775268.1		
	'*Ca.* P. fragariae''isolate GBFC SY 03	MN907121.1		
	Corylus avellana proliferation phytoplasma strain 21419156	KP407881.1		
	'*Ca.* P. fragariae'	EF015582.1		
	'*Ca.* P. fragariae' clone GBFC SY 01	MK501641.1		
	Solanum tuberosum stolbur phytoplasma strain Ningxia	MK696087.1		
	'*Ca.* P. fragariae' strain CN-159	HQ599235.1		
	'*Ca.* P. fragariae' strain CN-4A	HQ609492.1		
	'*Ca.* P. fragariae' strain YN-2G	EU293841.1		
	'*Ca.* P. fragariae' clone GBFC SY 02	MK501642.1		
	'*Ca.* P. fragariae' isolate GBFC SY 04	MN907122.1		
	'*Ca.* P. fragariae' strain CN-89	HQ599236.1		
	'*Ca.* P. fragariae' isolate GBFC SY 06	MN907124.1		
	'*Ca.* P. fragariae'	MH061346.1		
	'*Ca.* P. fragariae' isolate GBFC SY 05	MN907123.1		
	'*Ca.* P. fragariae'	DQ086423.1		
	Cordyline phytoplasma	EU168792.1		
	'*Ca.* P. fragariae' strain StrawY	HM104662.1		
Americanum' Cluster	'*Ca.* P. americanum' strain PPT10-NE	DQ174121.1		
	'*Ca.* P. americanum' isolate SRL1-PA1	MN227133.1		
	'*Ca.* P. americanum' strain PPT1-TX	DQ174118.1		
	'*Ca.* P. americanum' strain PPT9-NE	DQ174120.1		
	'*Ca.* P. americanum' strain PPT12-NE	DQ174122.1		
	'*Ca.* P. americanum' clone 1863	OK033873.1		
	'*Ca.* P. americanum' clone 1891	OK033874.1		
Convolvuli Cluster	Carica papaya bunchy top phytoplasma isolate NGPBT-12	MW530522.1		
	Bindweed yellows phytoplasma strain GE27	KF996537.1		
	'*Ca.* P. convolvuli' strain BY-S57/11	JN833705.1		
	'*Ca.* P. convolvuli' clone Rus217	OQ103122.1		
	'*Ca.* P. convolvuli' strain BY-BH1	JN833708.1		
	'*Ca.* P. convolvuli' strain BY-S62/11	JN833706.1		
	'*Ca.* P. convolvuli' strain BY-BH2	JN833709.1		
	'*Ca.* P. convolvuli' strain BY-I1015	JN833710.1		
	'*Ca.* P. convolvuli' strain BY-I1016	JN833711.1		
	'*Ca.* P. convolvuli' strain BY-G	JN833707.1		
	'*Ca.* P. convolvuli' isolate 14-19	MG564476.1		
	'*Ca.* P. convolvuli' clone Ca 21-8-18	MW037215.1		
	Bindweed yellows phytoplasma	Y16391.1		
Japonicum Cluster	'*Ca.* P. japonicum'	AB010425.2		
	'*Ca.* P. japonicum' strain JHP	AB639071.1		
	'*Ca.* P. japonicum' strain JHP	AB639072.1		

Stolbur genomes analysed are indicated in bold.

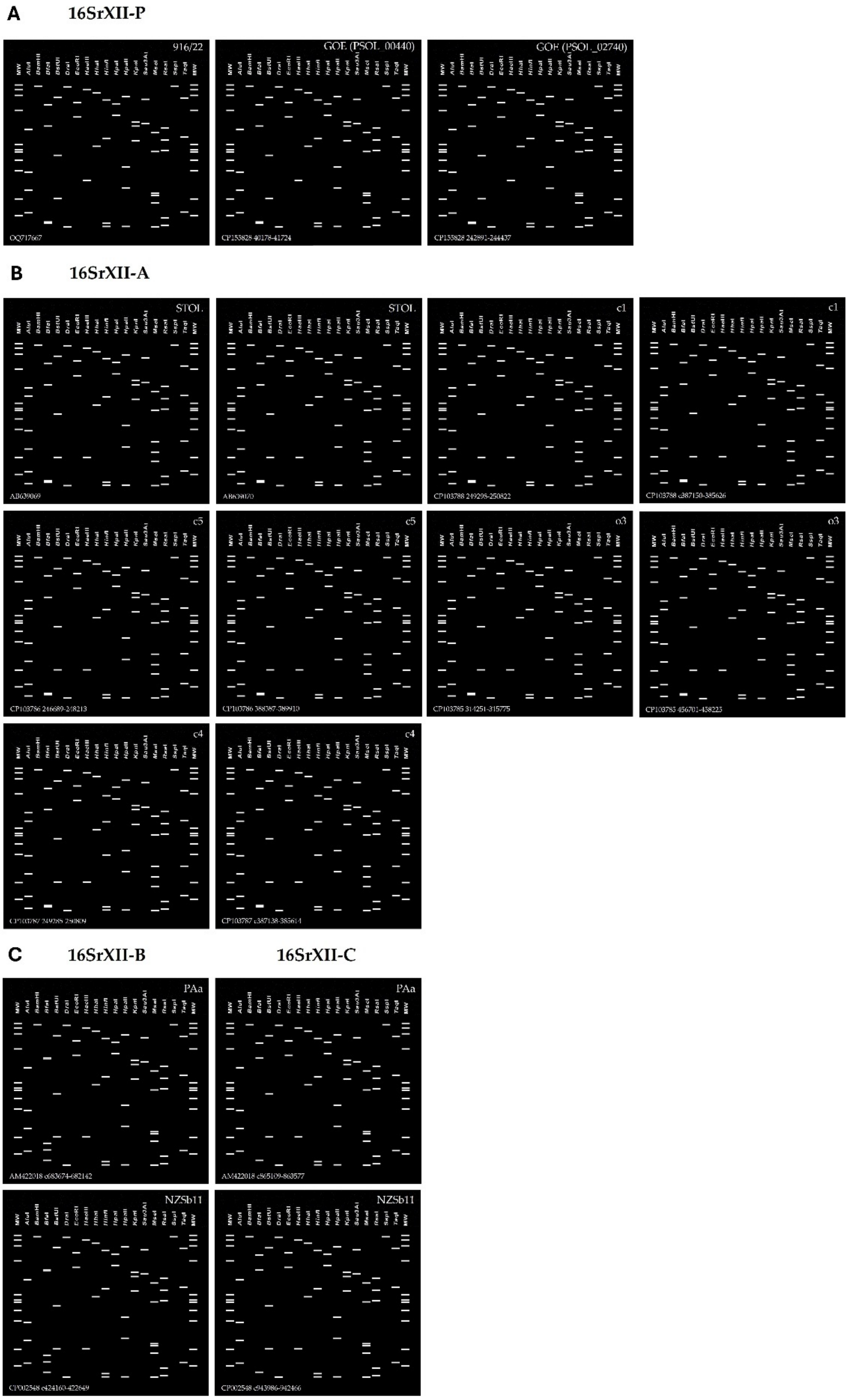

Figure S1. Virtual gel images of the RFLP analysis of the analysed stolbur genomes and representative reference strains obtained from the digestion with all 17 key restriction enzymes. The gels are grouped according to their 16SrXII subgroup affiliation as follows: (A) 16SrXII-P, (B) 16SrXII-A, and (C) 16SrXII-B/-C. Corresponding accessions and positions in the genome sequences are indicated at the bottom of each virtual gel image.

Table S2. Unique deduced amino acid sequences of 16SrXII-P phytoplasma strain GOE.

Locus tag	Product
**Species specific**	
PSOL_00650	DUF2963 domain-containing protein
PSOL_00660	hypothetical protein
PSOL_00690	putative membrane protein
PSOL_00770	hypothetical protein
PSOL_01590	putative membrane protein
PSOL_01620	hypothetical protein
PSOL_01630	DUF2963 domain-containing protein
PSOL_01770	hypothetical protein
PSOL_02000	ATP-dependent zinc metalloprotease, FtsH
PSOL_03110	hypothetical protein
PSOL_03340	hypothetical protein
PSOL_03590	hypothetical protein
PSOL_03600	DUF2963 domain-containing protein
PSOL_04350	hypothetical
PSOL_04370	hypothetical
PSOL_04430	hypothetical
PSOL_04490	hypothetical
PSOL_04510	hypothetical
PSOL_04540	ATP-dependent Zn protease
PSOL_04550	putative membrane protein
PSOL_05410	hypothetical protein
PSOL_06240	hypothetical protein
PSOL_06790	hypothetical protein
PSOL_06930	hypothetical protein
PSOL_07030	putative secreted protein
PSOL_07160	hypothetical protein
PSOL_07310	putative membrane protein
**Unassigned**	
PSOL_00190	putative membrane protein
PSOL_00250	putative membrane protein
PSOL_00270	hypothetical protein
PSOL_00360	hypothetical protein
PSOL_00370	hypothetical protein
PSOL_00400	hypothetical protein
PSOL_01140	hypothetical protein
PSOL_01150	hypothetical protein
PSOL_01220	putative secreted protein
PSOL_01290	hypothetical protein
PSOL_01740	hypothetical protein
PSOL_01800	putative membrane protein
PSOL_02020	putative membrane protein
PSOL_02040	putative membrane protein
PSOL_02060	hypothetical protein
PSOL_02110	hypothetical protein
PSOL_02150	hypothetical protein
PSOL_02190	hypothetical protein
PSOL_02210	hypothetical protein
PSOL_02220	hypothetical protein
PSOL_02230	hypothetical protein
PSOL_02270	putative membrane protein
PSOL_02280	putative membrane protein
PSOL_02300	putative membrane protein
PSOL_02310	hypothetical protein
PSOL_02320	hypothetical protein
PSOL_02480	putative membrane protein
PSOL_02490	hypothetical protein
PSOL_03430	hypothetical protein
PSOL_03730	variable membrane protein
PSOL_03800	hypothetical protein
PSOL_03830	hypothetical protein
PSOL_04040	putative membrane protein
PSOL_04090	putative membrane protein
PSOL_04160	hypothetical protein
PSOL_04410	hypothetical protein
PSOL_04530	hypothetical protein
PSOL_04610	putative membrane protein
PSOL_04790	hypothetical protein
PSOL_04820	hypothetical protein
PSOL_04880	hypothetical protein
PSOL_04890	putative secreted protein
PSOL_05270	hypothetical protein
PSOL_05300	putative membrane protein
PSOL_05400	putative membrane protein
PSOL_05420	hypothetical protein
PSOL_05430	hypothetical protein
PSOL_05460	putative membrane protein
PSOL_05470	hypothetical protein
PSOL_05620	ABC-type lipoprotein transport system, ATP-binding protein/permease protein
PSOL_05640	hypothetical protein
PSOL_05810	putative secreted protein
PSOL_05840	putative membrane protein

PSOL_05850	putative membrane protein
PSOL_05980	hypothetical protein
PSOL_06030	hypothetical protein
PSOL_06040	hypothetical protein
PSOL_06050	hypothetical protein
PSOL_06070	hypothetical protein
PSOL_06110	hypothetical protein
PSOL_06150	putative membrane protein
PSOL_06180	putative membrane protein, SVM family protein
PSOL_06200	hypothetical protein
PSOL_06210	putative membrane protein
PSOL_06320	putative membrane protein
PSOL_06380	hypothetical protein
PSOL_06390	putative membrane protein
PSOL_06460	putative membrane protein
PSOL_06570	hypothetical protein
PSOL_06610	putative membrane protein
PSOL_06760	putative secreted protein
PSOL_06770	hypothetical protein
PSOL_07010	putative membrane protein
PSOL_07080	hypothetical protein
PSOL_07300	hypothetical protein
PSOL_07340	putative membrane protein

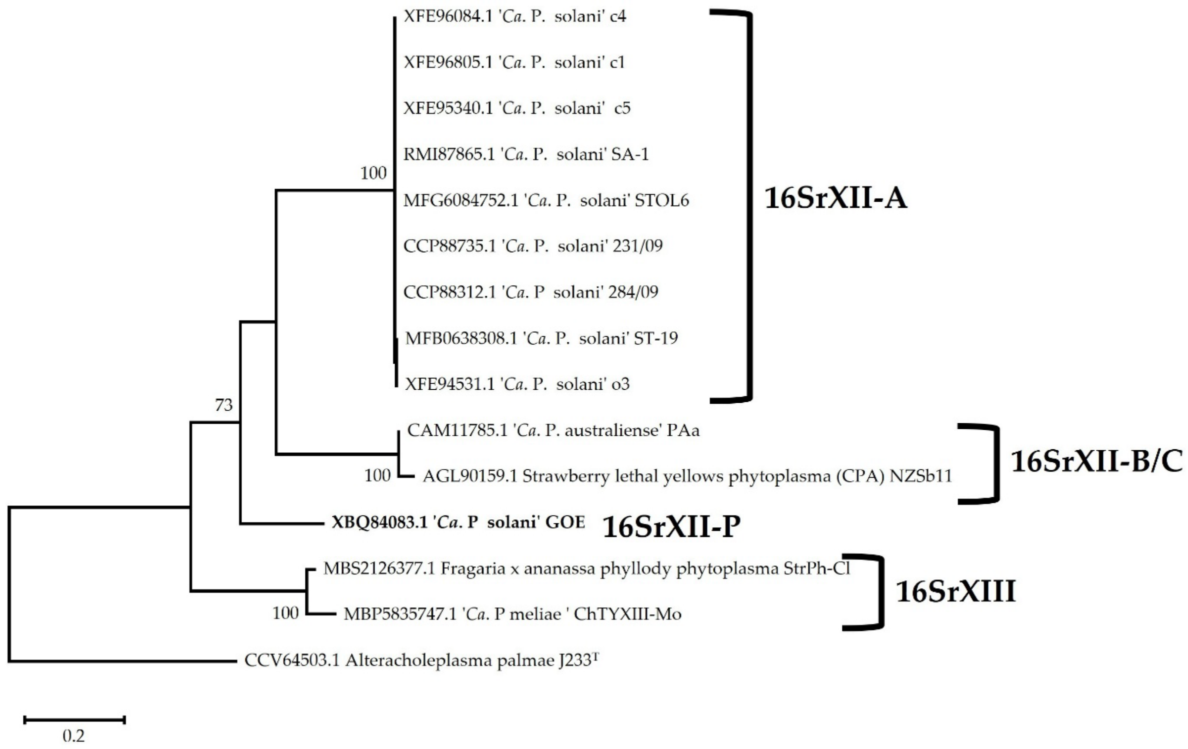

Figure S2. Maximum likelihood phylogeny based on the gene *ribF*. Names of strains with their respective GenBank accession are given. 16Sr group/subgroup affiliation is highlighted next to the clusters. Only bootstrap support values of 70 or above are displayed, with data obtained from 1,000 replicates. Scale bars indicate substitutions per site.

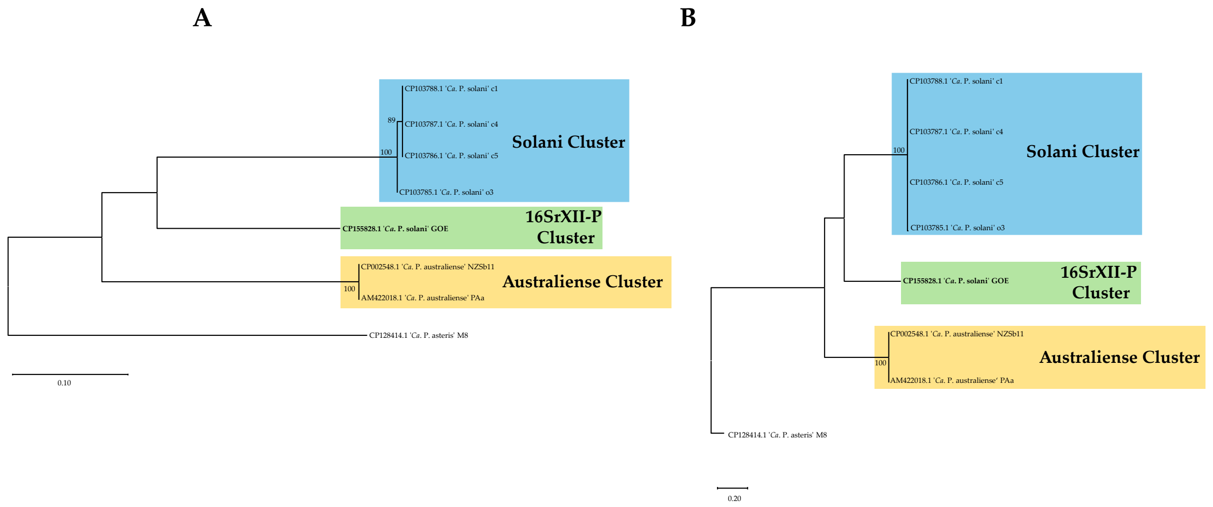

Figure S3. Maximum likelihood phylogeny of the analysed stolbur genomes based on the gene *imp* (A) and the deduced amino acid sequences of Imp (B). Names of strains with their respective GenBank accession are given. Only bootstrap support values of 70 or above are displayed, with data obtained from 1,000 replicates. Scale bars indicate substitutions per site.
